# The Role of Myeloid-Derived Suppressor Cells in Patients with Solid Tumors: A Meta-Analysis

**DOI:** 10.1371/journal.pone.0164514

**Published:** 2016-10-25

**Authors:** Shuo Zhang, Xuelei Ma, Chenjing Zhu, Li Liu, Guoping Wang, Xia Yuan

**Affiliations:** State Key Laboratory of Biotherapy and Cancer Center, West China Hospital, Sichuan University, Chengdu, PR China; Universidad de Palermo, UNITED STATES

## Abstract

Targeting immune cells or factors are effective for patients with solid tumors. Myeloid-derived suppressor cells (MDSCs) are known to have immunosuppressive functions, and the levels of MDSCs in patients with solid tumor are assumed to have prognostic values. This meta-analysis aimed at evaluating the relationship between MDSCs and the prognosis of patients with solid tumors. We searched articles in PUBMED and EMBASE comprehensively, updated to March 2016. Eight studies with 442 patients were included in the meta-analysis. We analyzed pooled hazard ratios (HRs) for overall survival (OS), disease-free survival (DFS) and progression-free survival (PFS). The results showed that MDSCs were associated with poor OS (HR, 1.94; 95% confidence interval [CI], 1.42–2.66; P < 0.0001) in patients with solid tumors. PFS/RFS (HR, 1.85; 95% CI, 1.16–2.97; P = 0.01) also indicated the association between MDSCs and prognosis. The HRs and 95% CIs for OS in Asian and non-Asian patients were 2.53 (95% CI 1.61–3.42, p < 0.00001) and 1.67 (95% CI 1.14–2.46, p < 0.0001), respectively. We further analyzed the data according to tumor types. The combined HRs and 95% CIs for OS were 1.26 (95% CI 1.10–1.44, p = 0.0003) for gastrointestinal (GI) cancer, 2.59 (95% CI 1.69–3.98, p < 0.0001) for hepatocellular carcinoma (HCC) and 1.86 (95% CI 1.26–2.75, p = 0.002) for other tumor types. In conclusion, MDSCs had a fine prognostic value for OS and PFS/RFS in patients with solid tumors. MDSCs could be used as biomarkers to evaluate prognosis in clinical practice.

## Introduction

The incidences of various solid tumors such as gastrointestinal (GI) cancer and breast cancer (BC) are increasing every year [1] and solid tumors are regarded as one of the most frequent causes of death worldwide [2–6]. Current therapies for different solid tumors include surgical resection, chemotherapy, radiotherapy and immunotherapy [1, 7, 8]. Recently, targeted immunotherapy such as cancer vaccines and monoclonal antibodies has been demonstrated to improve anti-tumor immune responses and may be beneficial for patients with different types of cancers [9, 10]. However, it was reported that cancer-related immune-suppression restricts the effects of immunotherapy [11].

Currently, it has been demonstrated that immune cells in tumor microenvironment help to form an immunosuppressive network, which plays a key role in the suppression of antitumor immune system, and finally leads to tumor invasion [12–14]. Myeloid-derived suppressor cells (MDSCs) are regarded as a heterogeneous population of immature myeloid cells with CD11b+CD33+HLA-DR−/low phenotype, which include granulocytic CD14−CD15+ and monocytic CD14+CD15− subtypes [15–17]. MDSCs, as immunosuppressive cell subjects, have been reported to play a critical role in mediating immune suppression by inhibiting both the innate and adaptive immunity [18–20] and preventing anticancer immunity function of cancer vaccines [13, 14, 21]. MDSCs inhibit the functions of immune cells through activating oxygen species The role of myeloid cells in the promotion of tumour angiogenesis [16, 17] and producing cytokines such as IL-6 and IL-4. Recently, some researches have reported that MDSCs limit the accumulation of T cells in both mice and human models with various cancers such as HCC [22, 23]. Therefore, targeting MDSCs which potentially stimulate anti-tumor immune system [24] may improve the effects of anti-cancer therapies [17, 25].

MDSCs are thought to have prognostic significance in patients with solid tumors [14, 26, 27]. At present, there is a heated controversy of MDSC on its prognostic significance. Thus a meta-analysis to investigate the prognostic value of MDSCs in patients with solid tumors is urgent.

## Materials and Methods

### Search Strategy

We performed a comprehensive search in PUBMED and EMBASE databases for all available studies published up to March 2016 to evaluate the prognostic value of MDSCs in patients with solid tumors. We used the following search terms: “Myeloid-derived suppressor cells OR MDSC” and “prognosis OR prognostic OR survival OR outcome” and “cancer OR tumor OR carcinoma OR neoplasm”. We also manually scanned the references of included articles in order to check more relevant studies. Our study was performed based on the Preferred Reporting Items for Systematic Reviews and Meta-Analyses (PRISMA) statement [28].

### Inclusion and Exclusion Criteria

The eligible studies in this meta-analysis must meet the following inclusion criteria: (1) published in English, (2) investigated patients with solid tumor, (3) contained information of level of MDSCs, (4) estimated the relationship between MDSCs level and survival outcomes. Articles were excluded with any of the following features: 1) studies without enough information to estimate HR and 95% CI; 2) studies had duplicate or overlapping data; 3) studies were not demonstrated in English.

### Data Extraction

Two reviewers extracted the required data from all available studies independently. Titles and abstracts were reviewed to identify potential available articles, and full texts were obtained for more details. We extracted the following contents: the first author’s name and country, publication year, number of patients, subtypes of MDSCs, cut-off value, survival analysis and the HRs of MDSCs for OS and PFS/RFS. If the HR and its 95% CIs could not be obtained directly, they were estimated from the corresponding data or Kaplan-Meier curves extracted from the studies according to the methods reported by Parmar et al [29]. Any discrepancies were resolved by consulting with a third author until consensus was reached.

### Statistical Analysis

HRs and the corresponding 95% CIs were calculated to estimate the association between MDSCs and patients’ prognosis according to Tierney’s method [30]. The heterogeneity of combined HRs was evaluated by Cochran’s Q test and Higgin’s I^2^ statistics [31, 32]. A P value < 0.05 and/or I^2^ >50% [31] indicated substantial heterogeneity among studies, and a random-effect model was used (DerSimoniane-Laird method) to calculate the combined HR; otherwise, a fixed-effect model (Mantel-Haenszel method) was used [33]. Because elements such as tumor types, region, number of patients and cut-off value may affect outcomes of this meta-analysis, we performed subgroup analyses. In general, if the 95% CI for the combined HR did not overlap one, pooled HR > 1 was thought to suggest a significant relationship with poor prognosis. A sensitivity analysis was conducted by deleting one study at a time to examine its effect on the pooled results. Publication bias was estimated using funnel plots qualitatively with the standard error [34], and evaluated by Begg’s and Egger’s test. All the analyses were carried out by STATA statistical software version 11.0 (StataCorp LP, College Station, TX, USA) and Review Manager Version 5.0 (Copenhagen: The Nordic Cochrane Centre: The Cochrane Collaboration, 2008).

### Quality Assessment

According to the nine-star Newcastle-Ottawa scale (NOS) [35], the quality of each study was strictly assessed in three aspects: selection (four points), comparability (two points), and outcome assessment (three points). A nine-point score is regarded as the highest score. Based on quality assessment standards from published meta-analyses [36], a trial with five or more points was identified as high quality. Articles with less than five points will not be retrieved in order to ensure the quality of the meta-analysis. Any ambiguity or differences in quality evaluation were reviewed and solved together by two authors.

## Results

### Study Selection and Characteristics

**Fig 1:**
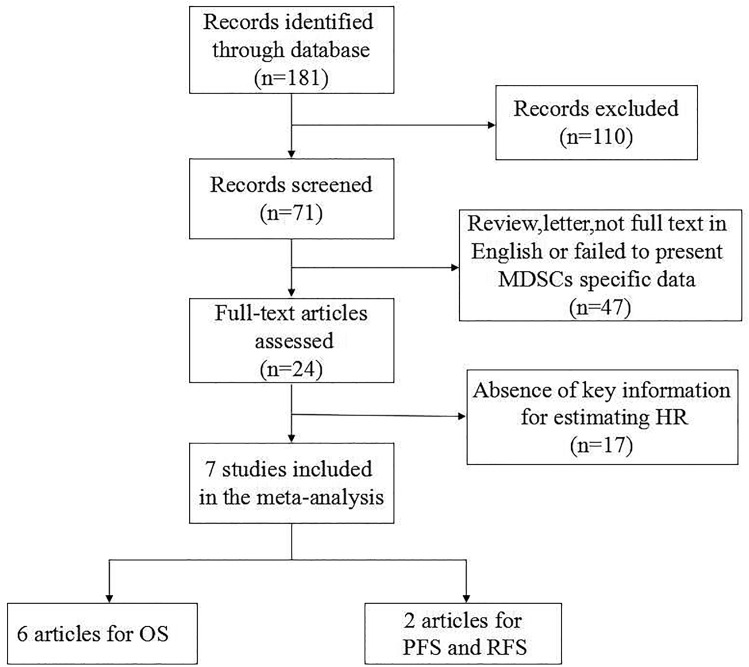
Methodological flow diagram of the meta-analysis.

As is shown in Fig 1, a total of 181 potentially relevant studies were retrieved according to the search methods. After the evaluation of titles and abstracts manually, forty-seven articles were excluded for the reasons shown in Fig 1. Full-text articles of the remaining 24 were assessed and 17 articles were further excluded due to the lack of essential data for estimating HR. Finally, a total of 7 studies including 442 patients were available for the meta-analysis.

Additionally, two articles [37, 38] studied the prognosis of patients before and after therapy. One [39] investigated two different types of cancer which were colorectal cancer and breast cancer and we retrieved the data of the two tumors separately.

The main features of the eligible studies were summarized in Table 1. The total number of patients was 442, ranging from 25 to 131 in each study. In total, OS data were available from 6 trials [13, 37, 39, 40], and 3 studies had data for PFS or RFS [40].

**Table 1:** Summary table of the meta-analysis.

Author	Year	Origin of population	Number of patients	follow-up (months)	MDSCs subtypes	Type	Cut-off	Sample collection	Survival analysis	HR(95%CI)
Arihara (OS) [33]	2013	Japan	33	NA	CD14^+^HLA-DR^-/low^	HCC	22%	PBMCs	OS	2.67 (1.29–5.52),
Arihara (RFS) [33]	2013	Japan	33	NA	CD14^+^HLA-DR^-/low^	HCC	23%	PBMCs	RFS	1.94 (1.17–3.21)
Gabitass [8]	2011	UK	131	NA	HLA-DR- Lin1^low/-^ CD33^+^ CD11b^+^	GI cancers	2%	PBMCs	OS	1.22(1.06–1.41)
Wang (Pre-therapy) [32]	2016	China	92	NA	CD14+HLA-DR−/low	HCC	14.60%	PBMCs	OS	2.257 (1.035–4.924)
Wang (Post-therapy) [32]	2016	China	92	NA	CD14^+^HLA-DR^−/low^	HCC	14.60%	PBMCs	OS	2.838 (1.379–5.837)
Weide [36]	2013	Australia	94	15	CD14^+^CD11b^+^HLA-DR^-/low^	advanced melanoma	11%	PBMCs	OS	1.7 (1.1–2.7)
Wang [37]	2012	Singapore	40	NA	Lin- HLADR^low^CD14^low/-^CD15^+^CD11b^+^CD33^+^	GC	4%	PBMCs	OS	1.69 (0.77–3.72)
Solito (CRC) [34]	2011	Italy	25	NA	Lin-/ HLA-DR-/ CD33+/ CD11b+	CRC	2.54%	PBMCs	OS	2.63 (1.15–5.98)
Solito (BC) [34]	2011	Italy	25	NA	Lin-/ HLA-DR-/ CD33+/ CD11b+	BC	3.17%	PBMCs	OS	2.73(1.12–6.66)
Tarhini [38]	2014	France	27	17.6	Lin1-/HLA-DR-/CD33+/CD11b+%	advanced melanoma	NA	PBMCs	PFS	1.37(0.37–5.26)

**Abbreviations:** OS, overall survival; NR, not reported; PFS, progression-free survival; RFS, recurrence-free survival; HCC, hepatocellular carcinoma; HL, hodgkin lymphoma; GC, gastric cancer; GI, gastrointestinal cancer; BC, breast cancer; CRC, colorectal cancer; PBMCs, Peripheral blood mononuclear cells

### Overall Survival

**Fig 2:**
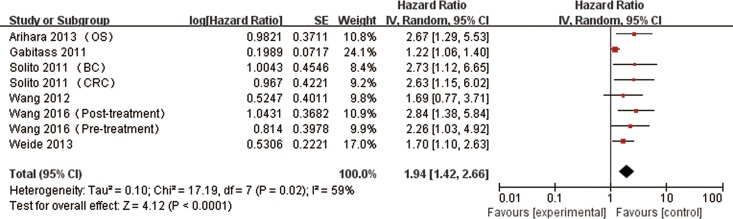
Meta-analysis of the association between MDSCs and OS in patients with solid tumors. Results are presented as individual and pooled hazard ratio (HR) with 95% confidence intervals (CIs) using a random-effect model.

**Fig 3:**
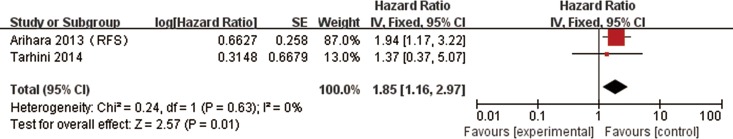
Meta-analysis of the association between MDSCs and PFS/RFS. Results are presented as individual and pooled hazard ratio (HR) with 95% confidence intervals (CIs) using a fixed-effect model.

The association between MDSCs and prognosis was shown in Figs 2 and 3. In total, elevated MDSCs predicted poor outcomes in patients with solid tumors. The combined HR was 1.94 (95%CI: 1.42–2.66, P < 0.0001) for OS with a random-effect model due to the significant heterogeneity (I^2^ = 59%, P = 0.02). PFS/RFS was 1.85 (95%CI: 1.16–2.97, P = 0.01) calculated using a fixed model (I^2^ = 5%, P = 0.35).

### Subgroup Analysis

To explore the origin of heterogeneity, subgroup analyses for OS were preformed based on tumor types, region, number of patients (number < 50 or ≥50) and cut-off value (cut-off ≥10% or ≤10%). The results (see Table 2) showed that HRs and 95% CIs for OS in GI cancer, HCC and other types of tumors were 1.26 (1.10–1.44), 2.59 (1.69–3.98) and 1.86 (1.26–2.75), respectively. The HRs and 95% CIs for OS in patients <50 group was 2.317 (1.59–3.54) and 1.70 (1.15–2.50) in patients ≥ 50 group. In the cut-off ≥10% group the HR was 2.11 (1.55–2.86) and in cut-off <10% group was 1.72 (1.09–2.71). It showed that MDSCs had a stronger prognostic value for OS in cut-off ≥10% group. In addition, we grouped the studies by patients’ ethnicity, and the HRs and 95% CIs for OS in Asia and non-Asia areas were 2.53 (1.61–3.42) and 1.67 (1.14–2.46), respectively.

**Table 2:** Stratified analyses of MDSCs on overall survival in patients with solid tumors.

Stratified analyses	Number of studies	Number of patients	Model	Pooled HR(95%CI)	I^2^	p-value
Tumor types						
GI cancers	4	196	Fixed	1.26(1.10–1.44)	47%	0.15
HCC	3	217	Fixed	2.59(1.69–3.98)	0%	0.91
Other types	2	146	Fixed	1.86(1.26–2.75)	0%	0.35
Region						
Asian	4	257	Fixed	2.53(1.61–3.42)	0%	0.79
Non Asian	5	322	Random	1.67(1.14–2.46)	61%	0.05
Number of patients						
≥50	4	409	Random	1.70(1.15–2.50)	65%	0.03
<50	5	150	Fixed	2.37(1.59–3.54)	0%	0.81
Cut-off						
≥10%	4	311	Fixed	2.11(1.55–2.86)	0%	0.57
<10%	5	221	Random	1.72(1.09–2.71)	55%	0.09

### Publication Bias and Sensitivity Analysis

**Fig 4:**
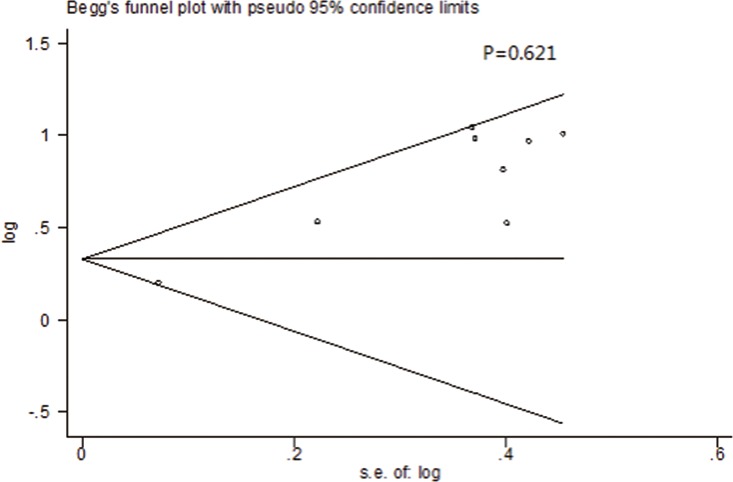
Summary of Begg’s funnel plots of publication bias for OS in all patients.

Both Begg’s funnel plot and the Egger’s test were conducted to assess the publication bias of included trials. As is shown in Fig 4, the shape of the funnel plots presented no significant asymmetry. The P values of Begger’s test for OS and PFS/RFS were 0.621 and 0.317, respectively, revealing that no obvious publication bias existed in this study. In order to evaluate the impact of each individual study, we conducted the sensitivity analysis on the pooled HRs for the OS or PFS/RFS. It turned out that there were no significant effects of individual study on the combined HRs. The results indicated that the outcomes of this meta-analysis were reliable.

## Discussion

Up to now, no meta-analysis has been conducted to assess the prognostic significance of MDSCs. Our combined results demonstrated that elevated MDSCs had a poor outcome in cancer patients.

MDSCs as a significant immune regulator suppress both innate and adaptive immunity and accelerate tumor progression [17, 41]. Due to their remarkable roles in the inhibition of resistance to clinical therapies, it has been assumed that limiting MDSC-mediated immunosuppression may activate antitumor immune response [42]. Some studies have demonstrated that through depletion of some amino acids such as arginine and cysteine, and improve inhibitory cytokines such as IL-10 and IL-12, MDSCs can cause suppression of immune cells and stimulate immune regulators such as tumor-associated macrophages (TAMs) [19, 41, 43, 44].

Considering the function of immunosuppressing, it seems that MDSCs play an important role in tumor growth and contribute to limiting the efficacy of anti-cancer therapies [17, 27, 45, 46]. Thus, it is conceivable that by helping tumor cells against immune system, MDSCs cause higher tumor relapse, deterioration and mortality [47]. There is growing evidence that higher MDSCs are associated with worse outcome. Some studies have evaluated the involvement of MDSCs in the progression of cancer patients, such as CRC [39], HCC [37, 38], GI cancers [13] and so on. What’s more, some experiments have found that targeting MDSCs can actually have positive effect on the angiogenesis of HCC [48, 49].

In our meta-analysis, there was obvious heterogeneity for OS (I^2^ = 59%, P = 0.02) among available studies. Therefore we adopted a random-effect model to calculate combined subgroup data. The heterogeneity may be caused by different features of included patients. In addition, different cancer types including HCC, CRC and so on may also contribute to the heterogeneity.

In order to find out causes of heterogeneity, subgroup analyses were conducted according to the country of patients, tumor types, number of patients and cut-off value. When we grouped the analysis based on tumor types, the heterogeneity for OS decreased. It implied that tumor types may contribute to heterogeneity. Also the HRs and 95% CIs for OS in patients in Asia and non-Asia were different, indicating that the prognostic role of MDSCs for OS of solid tumors was more significant in Asian group, and MDSCs also had a stronger prognostic value for OS with cut-off ≥10%. All the subgroup analysis had positive consequences.

There were some limits in this meta-analysis. First of all, the sample size of each type of cancers were relatively small, and more studies with large sample size were needed. Secondly, because some HRs could not be extracted directly from articles, we calculated them according to Kaplan-Meier survival curves which may make the results less reliable. Moreover, different cut-off value in the studies may also contribute to inter-study heterogeneity, but we carried out subgroup sensitivity analyses to overcome the shortcoming. Fourth, obvious heterogeneity existed because of different population characteristics or study designs. Finally, there may be some unavoidable bias because positive results tended to be published than negative ones, which may lead to the exaggeration of the correlation between MDSCs and poor prognosis. To avoid this, we examined the bias by excluding one study at a time.

In summary, results of this meta-analysis demonstrated that MDSCs were associated with poor prognosis in patients with solid tumor, and its prognostic role for OS was more significant in asian group. What’s more, MDSCs had a stronger prognostic value for OS with cut-off ≥10%. Therefore, MDSCs could be used as biomarkers to evaluate prognosis in clinical practice.

## Supporting Information

S1 Checklist PRISMA-IPD Checklist of items to include when reporting a systematic review and meta-analysis of individual participant data (IPD) [50]. (DOC)
